# Second-Chance Signal Transduction Explains Cooperative Flagellar Switching

**DOI:** 10.1371/journal.pone.0041098

**Published:** 2012-07-23

**Authors:** Henry G. Zot, Javier E. Hasbun, Nguyen Van Minh

**Affiliations:** 1 Department of Biology, University of West Georgia, Carrollton, Georgia, United States of America; 2 Department of Physics, University of West Georgia, Carrollton, Georgia, United States of America; 3 Department of Mathematics, University of West Georgia, Carrollton, Georgia, United States of America; German Cancer Research Center, Germany

## Abstract

The reversal of flagellar motion (switching) results from the interaction between a switch complex of the flagellar rotor and a torque-generating stationary unit, or stator (motor unit). To explain the steeply cooperative ligand-induced switching, present models propose allosteric interactions between subunits of the rotor, but do not address the possibility of a reaction that stimulates a bidirectional motor unit to reverse direction of torque. During flagellar motion, the binding of a ligand-bound switch complex at the dwell site could excite a motor unit. The probability that another switch complex of the rotor, moving according to steady-state rotation, will reach the same dwell site before that motor unit returns to ground state will be determined by the independent decay rate of the excited-state motor unit. Here, we derive an analytical expression for the energy coupling between a switch complex and a motor unit of the stator complex of a flagellum, and demonstrate that this model accounts for the cooperative switching response without the need for allosteric interactions. The analytical result can be reproduced by simulation when (1) the motion of the rotor delivers a subsequent ligand-bound switch to the excited motor unit, thereby providing the excited motor unit with a second chance to remain excited, and (2) the outputs from multiple independent motor units are constrained to a single all-or-none event. In this proposed model, a motor unit and switch complex represent the components of a mathematically defined signal transduction mechanism in which energy coupling is driven by steady-state and is regulated by stochastic ligand binding. Mathematical derivation of the model shows the analytical function to be a general form of the Hill equation (Hill AV (1910) The possible effects of the aggregation of the molecules of haemoglobin on its dissociation curves. J Physiol 40: iv–vii).

## Introduction

Bacterial flagellar switching is a process by which the rotation direction of a flagellum reverses. The switching process involves the interaction between a switch complex of the flagellar rotor and a torque-generating stationary unit, or stator. By regulating this interaction, the concentration of an intracellular signaling ligand, CheYP, determines flagellar switching behavior, including the frequency of switching, intervals of counterclockwise (CCW) and clockwise (CW) rotation, and average rotating time in the CW direction, referred to as CW bias [1]–[3]. Transitions between CCW and CW flagellar rotation occur stochastically [4]. However, the frequencies of switch intervals fit a gamma distribution [5], consistent with a switching process that includes a non-equilibrium step [6], which has been suggested to be related to steady-state flagellar rotation [7]. Thus, evidence supports both stochastic and deterministic steps in the process leading to a switching event.

Ligand CheYP binding to the flagellar rotor [8], [9] induce structural changes in the rotor that account for the change in motor direction. Reconstructions of electron micrographs reveal that the rotor is composed of approximately 34 repeating structures, called switch complexes, which are connected to each other to form a ring [10], [11]. Each switch complex is composed of two components: FliM, which has a binding site for a CheYP molecule, and FliG, which has a binding site for a torque-generating motor unit [10], [11]. Detailed structural analyses of the rotor have suggested that a ligand-induced conformational change in FliG is responsible for reversing the direction of flagellar rotation [12], [13].

In the process of switching, structural changes in the rotor could be transmitted to the stator. The stator comprises approximately 10 motor units, which are connected by the torque transmitted through the rotor [10], [11]. The motor units turn over rapidly, resulting in a dynamic stator organization [14]. The finding that flagellar rotation speed increases in proportion to the number of active motor units provides evidence for functional independence of the torque-generating units [15], [16]. At loads near zero, sequential activation of individual motor units produces an all-or-none jump to the maximum rotor speed, suggesting a high duty ratio for each motor unit and a single rate-limiting step for an ensemble of motor units acting on a common rotor [17]. Motor units of the stator are likely to react independently of the ligand-bound states of the rotor.

The binding of CheYP shifts the probability of the rotation direction in favor of CW rotation, i.e., CW bias [3], [18]. CW bias has a steep dependence on ligand concentration, with a Hill coefficient [19] of 10.3, suggesting an allosteric form of regulation [18], [20]. Allosteric regulation in this context refers to the stoichiometric conversion of all switch complexes to the ligand-bound conformation as a result of sub-stoichiometric binding site occupancy. The simplest version of concerted allosteric regulation proposes that all switch subunits of the rotor change conformation in unison [18]. If the conformational change in the rotor were to be concerted, the stator would receive a binary signal, making the transmission step between the rotor and stator trivial. With allosteric regulation, the direction of the motor would not need to change, but rather a unidirectional motor could drive a ratchet-like mechanism in either direction.

CheYP has been shown to bind non-cooperatively to the switch complexes of a rotor [21], [22], consistent with independent, non-interacting ligand binding sites. The ligand binding data are most consistent with a version of allosteric regulation whereby a conformational change propagates between ligand-bound switch complexes of the rotor (conformational spread) [23], [24]. Although the conformational spread model requires less cooperative ligand binding compared with the concerted model [23], [24], the CheYP binding site occupancy predicted by this model [24] is not consistent with the measured occupancy of switch complexes [21], [22]. Theoretically, a steady-state process can propagate an analogous conformational change in the rotor without requiring positive cooperativity of ligand binding [6], [7]. However, the application of these findings awaits direct evidence for a structural change of the rotor subunits brought about by interaction with the stator.

Consistent with the binding data, we suggest in this paper that flagellar switching is regulated by stochastic ligand binding and that a cooperative response is generated without an allosteric mechanism. To develop a mathematical model of the flagellar switching mechanism, we start with a model based on the regulation of vertebrate striated muscle, which has been shown to generate cooperative responses from non-cooperative ligand binding [25]. Although muscle and flagella have no structural components in common, a unifying physical characteristic may underlie both systems, and this may be mathematically deduced from the model representing the structure-function relationship of muscle [25],
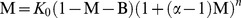
(1.1)where M and B represent excited and relaxed states of tropomyosin, respectively; *n* is the ensemble number of tropomyosin subunits; *K*_0_ is an apparent constant; and *α* is a parameter with no defined physical significance. In the muscle system, tropomyosin and myosin behave respectively as a reader, which detects a signal, and a switch, which provides a signal. Its interaction with myosin drives tropomyosin to the M state. Here, we derive a strikingly similar mathematical form for the regulation of flagellar switching based on the unique structural features of the flagellar motor. The interaction between a flagellar motor unit and a switch complex stimulates the motor unit to an excited state capable of switching the direction of the torque. The results described here provide a biochemical definition for *α* and a framework for a generalized model of cooperativity based on non-equilibrium, rather than allosteric, biochemistry.

## Methods

### Description of the Model

**Figure 1:**
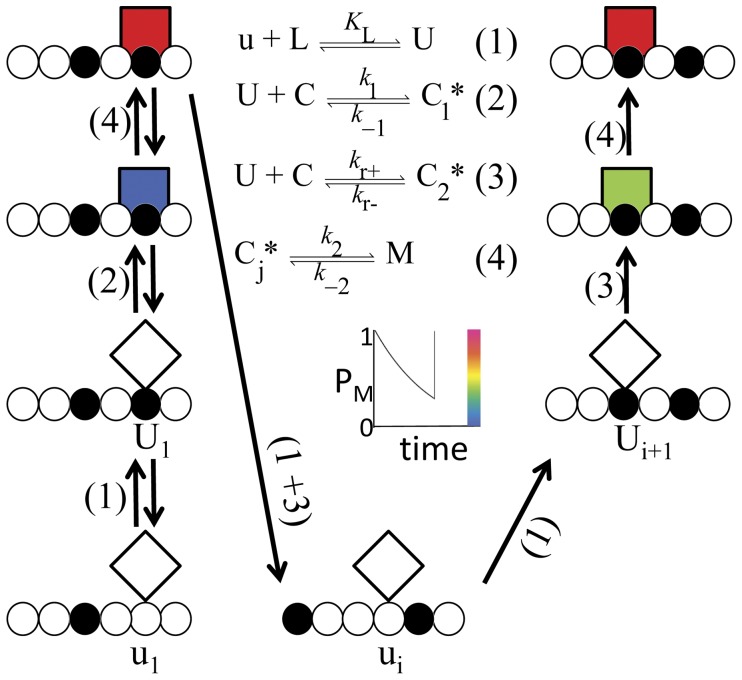
Second-chance scenario for a bacterial flagellar motor unit. The diagram shows the pathways by which traveling switch components of the rotor (circles) stimulate a motor unit (square sides). At time zero, the motor in the ground state (C) has the null probability of being excited (blue). Formation of a collision complex (C_j_*; j = 1,2) stimulates a motor unit to an excited state (M) with probability (P_M_) of unity (red). While P_M_ decays (color key in inset), the rotor traveling at a constant rate, *k*_r_, breaks contact with one switch complex (U_1_) at rate *k*_r−_ = *k*_r_ and delivers another switch complex (U_i_, i>1) to the motor unit at rate *k*_r+_ = *k*_r_. The ligand concentration, [L], determines whether a switch can form a collision complex (filled circle; U) or not (open circle; u). If by chance, θ = *K*_L_[L]/(1+*K*_L_[L]), the switch complex at the dwell site is occupied by ligand, and the formation of C_j_* restores P_M_ to unity. An equilibrium pathway is required to initiate motor unit excitation (Eq. 1, 2, and 4; Fig. 1). The second-chance pathway (Eq. 1, 3, and 4; Fig. 1) can sustain the excited state. To emphasize the pathway-dependent free energy of the collision complex, the subscripts in C_1_* and C_2_* are included in the symbols for intermediate products of the equilibrium and second-chance pathways (Reactions 2 and 3), respectively. It should be noted that C_1_*, C_2_*, and C_j_* represent the same change in physical structure of the motor unit as is represented by the transition between diamonds and squares in the schematic. To recapitulate, temporal changes in P_M_ may be traced for the scenario shown (inset). Decay of the excited state is by single exponential (inset). In a different scenario, had the motor unit returned to the ground state before being stimulated again, the equilibrium pathway would have been required to initiate a new excited state.

To model the flagellar switching system, we propose that a motor unit can exist in either a ground state (C) or an excited state (M) and that a switch complex can exist in either a ligand-free (u) or ligand-bound state (U) (Fig. 1). The ligand-bound switch forms via the interaction between the ligand-free state and CheYP ligand (L) by simple mass action (Reaction 1; Fig. 1). The motor unit transitions from ground state to excited state by interacting with the ligand-bound switch to form a collision complex, represented by C*. Each motor unit state, C, C*, and M (Fig. 1), has a defined unique structure, and only C* has pathway-dependent free energy. We use a composite symbol (C_j_*; j = 1, 2) to represent the intermediates of alternate reactions between the ground (C) and excited (M) states of the motor unit (Reactions 2 and 3, Fig. 1). Thus, C_1_* and C_2_* are transition states of an equilibrium pathway (Reactions 1, 2, and 4; Fig. 1) and a rotation-dependent pathway (Reaction 1, 3, and 4; Fig. 1), respectively. We refer to the rotation-dependent pathway as the second-chance pathway.

For modeling an arbitrary system, we use mole fraction quantities for u, U, C, and M, which are dimensionless quantities normalized to the total number of motor units in a given functional ensemble, *y*_tot_. Thus, given that *y*_tot_ is the sum of the numbers of ground and excited motor units (*y*_C_ plus *y*_M_), the ratios *y*_C_/*y*_tot_ and *y*_M_/*y*_tot_ are equal to the fractions of motors in the ground and excited states, C and M, respectively. Similarly, assuming a one-to-one interaction between a switch complex and a motor unit, the sum of ligand-free and ligand-bound switch complexes, *x*_u_ and *x*_U_, is *y*_tot_, and the ratios *x*_u_/*y*_tot_ and *x*_U_/*y*_tot_ are equal to u and U, respectively. The distribution of the switch complexes between the u and U states is determined by the ligand concentration, [L], according to mass action: U = *K*_L_[L]u, where *K*_L_ is the equilibrium constant for the reaction. As defined, u, U, C, and M have values between 0 and 1, and hence behave as probabilities for all *y*_tot_≥1. L has units of concentration.

At the moment of motor unit stimulation (time, *t* = 0), the probability that the excited state (P_M_) will occur is unity. After stimulation (*t*>0) and before the motor unit decays to the ground state, P_M_ can return to unity by either the equilibrium pathway or the second-chance pathways (inset; Fig. 1). Thus, although the equilibrium and second-chance pathways contribute independently to increasing the probability of the excited state, the second-chance pathway cannot stimulate a motor unit in the ground state.

The essential aspects of second-chance signal transduction can be seen by following the chance events of a single motor unit over time (Fig. 1). According to the model, a collision event C_j_* decays to the excited state M (Reaction 4; Fig. 1) with unitary probability (P_M_) at the moment of M formation (*t* = 0) (inset; Fig. 1). Subsequently, at *t*>0, another C_j_* decay can return P_M_ to unity (diagram and inset; Fig. 1); thus, M can be sustained by opportunities for C_j_* events. A sustaining C_j_* event could be generated by the equilibrium pathway (scenario not shown; Fig. 1) or by the rotation of the rotor (rotation-dependent second-chance pathway shown; Fig. 1), and the pathway choice is determined by chance. While a ground-state motor unit (C) can be stimulated only by the equilibrium pathway, the rotation-dependent pathway provides the excited state M an additional opportunity, or second chance, to be sustained.

### Derivation of the Analytical Function

To derive the rate of change in M by the equilibrium pathway, we define an arbitrary rotor subunit as u_1_. From Reactions 1, 2, and 4 (Fig. 1), U_1_ = *K*_L_[L]u_1_, C_1_* = *k*_1_U_1_ C, and M = *k*_2_ C_1_* which, by substitution, yields an expression, *k*_2_ *k*_1_ C*K*_L_[L]u_1_ for the forward rate of M. The reverse rate of M is given by the expression *k*_−2_ *k*_−1_M. Hence, the rate of change in the equilibrium pathway, *d*M_1_/*dt*, is given by
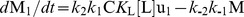
(2.1)Because the reaction that forms M from the ground state consumes U_1_ and C, the following conservation expressions must hold
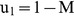
(2.2)

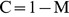
(2.3)The rotor rotates at a constant rate (*k*_r_). The rotation disrupts C_2_* at rate *k*_r−_ = *k*_r_. C_2_* forms with another switch, U_i_ (i>1), at rate *k*_r+_, which is limited by either the intrinsic reaction mechanism or the rate of the rotor. Hence, *k*_r+_≤*k*_r_.

From the rotation-dependent dissociation of C_2_* (Reaction 3; Fig. 1) and the independent decay of M (Reaction 4, Fig. 1), the reversal rate of M is given by *k*_−2_ *k_r−_*M. The rate of M stimulation is determined from U_i_ = *K*_L_[L]u_i_ (Reactions 1, Fig. 1), C_2_* = *k*_r+_U_i_C (Reaction 3, Fig. 1) and M = *k*_2_ C_2_* (Reaction 4, Fig. 1). Substitution, yields a single expression, *k*_2_ *k*_r+_C*K*_L_[L]u_i_ for the forward rate. Hence, the rate of change in the second-chance pathway, *d*M_2_/*dt*, is given by
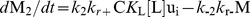
(2.4)The removal of U_1_ is canceled by the delivery of U_i_, and thus conservation is satisfied overall, and
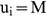
(2.5)From Eqs. 2.1 and 2.4, we see that the rate of change in each pathway is the sum of the functions of M. Hence, by the Sum Rule, the time derivative of M is,
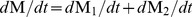
(2.6)By substituting Eqs. 2.1 and 2.4, Eq. 2.6 becomes
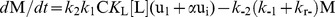
(2.7)where we define
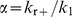
(2.8)At steady-state, *d*M/*dt* = 0, and Eq. 2.7 becomes
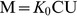
(2.9)with the further definitions
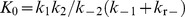
(2.10)and
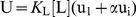
(2.11)Although the excited state of a given motor unit is given by Eq. 2.9, the torques of multiple motors should reverse when the rotational direction of the rotor switches. To account for the concerted action of *n* motor units, we generalize M to be the probability of CW rotation of all *n* motor units of an ensemble. To achieve a switch, we adjust the model so that the internal states of all *n* motor units coupled to the rotor must agree. This all or none requirement is expressed biochemically by the reaction
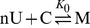
(2.12)Hence,
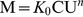
(2.13)By substituting Eqs. 2.2, 2.3, 2.5, and 2.11, Eq. 2.13 can be written as
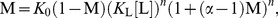
(2.14)which is used for calculating CW bias. We point to the similarity between this flagella behavior expression and that of muscle regulation, (Eq. 1.1), as mentioned earlier. Furthermore, it is interesting that this expression actually yields the Hill equation [19] for the special case when *α* = 1. As far as we know, this is the first time that such result has been demonstrated from first principles. When motion ceases, *k*_r−_ = *k*_r+_ = 0, *α* = 0, and only the equilibrium pathway exists.

### Simulation of Binary Switching Events

We devised a computer program (details in Methods S1) to simulate the stimulation of a motor unit by switches being moved by the rotor. In this program, chances for collision complex formation are a sequence of pulses generated at the rate of rotor rotation (Fig. S1). Each pulse has an adjustable duration corresponding to the time a switch dwells with a motor unit and amplitude corresponding to the probability of ligand occupancy (θ). Based on θ, a stochastic binding event is simulated as either ligand-bound or ligand-free (Fig. S2). If the switch is ligand-bound, the P_M_ is set to unity; if the switch is ligand-free, P_M_ = 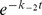, where *k*_−2_ is the rate of excited state decay and *t* is the time elapsed since the last excitation (Fig. S3). Based on P_M_, the program simulates a stochastic event (Fig. S4) corresponding to the output (C = 0 or M = 1). To simulate an all-or-none output of an ensemble of *n* motor units, the program compares the most recent dwell time state of the ensemble (CCW = 0 or CW = 1) to the present time outputs of individual motor units. If the outputs of all *n* motor units agree and the output disagrees with the most recent state of the ensemble, the program switches the state of the ensemble in the present time (Figs. S4, S5, S6, S7).

Details of the program are contained in the supplement (Methods S1). Preliminary simulations established the decay rates that correspond best to *α* = 1 and *α* = 2 (Fig. S8). Simulations were run with a constant sample time (1 unit/event) and arbitrary θ for 10,000 pulses.

## Results

**Figure 2:**
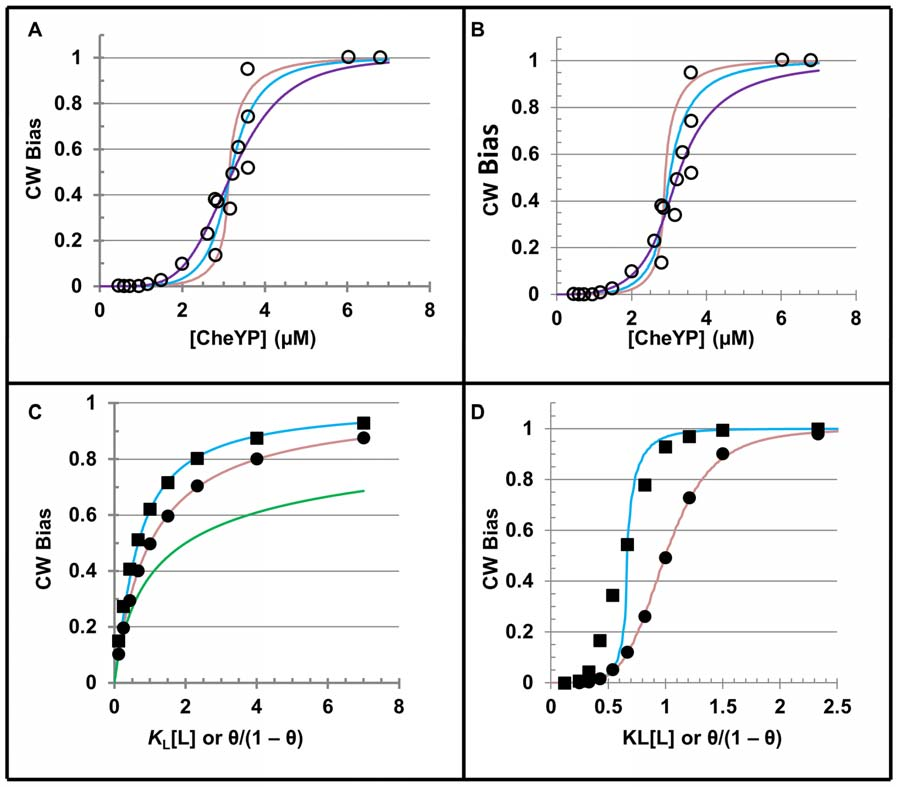
Model prediction compared with published and simulated data. **A and B.** Panels show the relationships of published data [20], [21] and representative plots using the analytical expression (Eq. 2.14) for arbitrary [CheYP] and *K*_L_ = 3.7 µM [21]. The parameters *α*, *K*_0_, and *n* of the analytical expression were adjusted to attain plots that were fit by appearance to the data. **Panel A.** We maintained constant *n* (*n* = 5) and varied *α* and *K*_0_. Shown are the fits for *α* and *K*_0_ given by 1 and 2 (purple), 1.5 and 0.7 (blue), and 2 and 0.3 (rose), respectively. **Panel B.** We maintained constant *α* and *K*_0_ (2 and 0.45, respectively) and varied *n*. Shown are the fits given *n* = 3 (purple), *n* = 4 (blue), and *n* = 5 (rose). **C and D.** Panels show relationships between plots of the M function (lines) and the results of simulation (symbols). Plots are generated as with Eq. 2.14 using *K*_0_ = 1, *K*_L_ = 1, and either *α* = 0 (green), *α* = 1 (rose), or *α* = 2 (blue). **Panel C.** *n* = 1. **Panel D.** *n* = 5. For the simulations, the rate of sample times and θ are as described in the *Methods* and the decay rates of the excited state (*k*_−2_) are 0.8 (squares) or 10 (circles), which were determined in preliminary measurements (details in Methods S1).

We propose that *α* depends on the number of switch complexes that reach the dwell site before the excited state of a motor unit has returned to ground state. To explore the influence of *α* on the output, we fit published data [20], taking a constant value of *n* = 5 in Eq. 2.14 (see discussion for rationale). The steepness of a curve depends directly on the value of *α*, as expected (Fig. 2A). Of interest is the finding that *K*_0_ must be adjusted upward as the value of *α* is reduced, in order to maintain a fit of the data. This implies that for the maintenance of constant coupling between a motor unit and switch complex, the coupling must become more effective as the opportunity for coupling decreases.

Increasing the value of *n* while holding *α* and *K*_0_ constant produces a more cooperative response (Fig. 2B). The data are not described best by any one curve, including the curve generated by the best fit with the Hill equation [20]. A positive trend in measured output at low ligand concentration fits the least cooperative model (*n* = 3), and the upward trend in gain at high ligand concentration fits the most cooperative model (*n* = 5). Taken together, these results suggest that the best model for describing the data may be one in which individual motor units are recruited to an ensemble as ligand is increased.

The analytical function (Eq. 2.14) is a macroscopic expression of underlying switching events taking place between individual molecules (Fig. 1). According to the model, the contribution of the second-chance pathway depends on the steady-state rate relative to the lifetime of the excited state, and a cooperative response can be produced by stochastic ligand binding.

To test these novel predictions, we devised a computer program that simulates stochastic binding events at the rate of the rotor using a pulse generator. We constrained the simulation by holding the outcomes of the internal events constant during the period between pulses, which reduces the model to the special condition, *k*_−1_ = 0 (Fig. 1). In effect, this prevents relaxation to the ground state via the equilibrium pathway and reduces the model to a rotation-dependent pathway, justifying the use of the pulse generator.

We also constrained the program to a single pathway. Combining the two pathways should not introduce systematic error when *α* = 1 because the equilibrium and second-chance pathways contribute equally to P_M_ in this special case of *α* (Eq. 2.8). By reducing program complexity, the program introduces error for *α*>1, but we more directly test the two novel predictions of the model, namely, *α* is a measure of rotor rate relative to motor unit decay and CW bias can be distributed cooperatively based on stochastic ligand binding.

We use high and low rates of excited state decay to simulate *α* = 1 and *α* = 2, respectively. At a high rate of decay, the simulation reproduces the output predicted for *α* = 1 (circles, Fig. 2C), consistent with the excited state receiving a second chance for stimulation before returning to ground state. When the decay rate is set low compared with the arbitrary dwell time, the simulated output approximates the output predicted for *α* = 2 of the analytical function (Eq. 2.14) (squares, Fig. 2C). This is consistent with the motor receiving two coupling opportunities, on average, to remain in the excited state. Although difficult to see in the plot (Fig. 2C), the predicted CW bias is over- or under-represented by the simulation with a low or high ligand concentration, respectively. A discrepancy is expected because the simulation treats the equilibrium and second-chance pathways as a single pathway, as described above. Had only the equilibrium pathway been simulated, the output would have been expected to approach the solution to the analytical expression for *α* = 0 (green, Fig. 2C).

**Figure 3:**
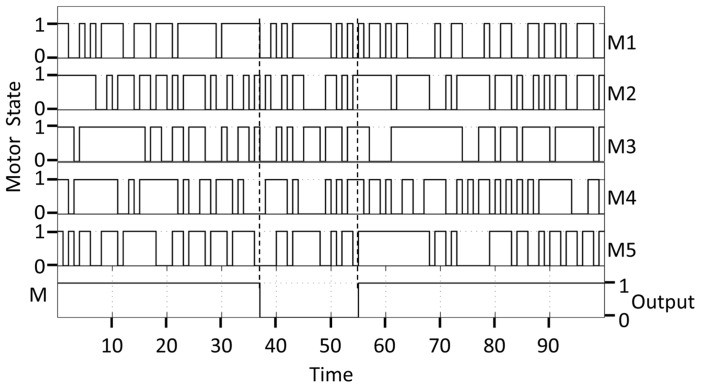
Ensemble output from five motor units. This figure demonstrates the criteria used by a computer program to simulate a switch in flagellar direction when multiple motor units act in a concerted manner on the same rotor. The record is a portion of that used in Fig. 2D for *α* = 2, *n* = 5, and θ = 0.6. The internal state of each of five motor units (M1–M5) can be excited (1) or ground (0) based on ligand binding. The output of the ensemble (M) switches from CW to CCW (0) or from CCW to CW (1) when the individual states of the motor units agree on bits 0 or 1 respectively. Dashed lines: a switch from CCW to CW occurs when the internal states of all five motor units are 1; a switch from CW to CCW occurs when the internal states of all five motor units are 0.

Given independent motor units, the excited states of an ensemble must be coordinated to achieve a uniform output from a given stator. To achieve an all-or-none output without altering the internal states of each motor unit of an ensemble, the simulated output reverses only when all motor units of the ensemble are in the same state (Fig. 3). The simulated binary events of the ensemble reverse much less often than the internal states of component motor units (Fig. 3). The simulation results for *n* = 5, *α* = 1 agree with the prediction (Fig. 2D). The obvious discrepancy between the simulation and prediction when *α* = 2 may be explained by the accumulation of errors inherent in the simulation of individual motor units, as described above. Hence, the results support a model in which individual motor units autonomously respond to switch complexes. A concerted output of a variable number of independent motor units can be achieved when the positive torque provided by even one motor unit overrides the tendency of individual motor units in an ensemble to reverse the sign of the torque.

## Discussion

Based on the flagellar system, we derived a relationship between ligand binding and CW bias (Eq. 2.14) that has the same form as a relationship obtained earlier [25] for a structurally and seemingly unrelated system of muscle (Eq. 1.1). A principal advance revealed by the flagellar system and reported here is that *α* is a steady-state parameter. We propose that steady-state events integrate with a standard equilibrium pathway to generate a cooperative response, given stochastic ligand binding.

The flagellar switching system may prove to be the clearest example of a generalizable biochemical mechanism. The motor unit and switch complex represent fundamental elements of the mechanism, namely, the reader and switch, respectively. The reader and switch form a collision complex that stimulates the reader to an excited state, which corresponds to CW torque of the flagellar motor unit. At the single molecule level, collision complex formation is an event that sets the probability of the excited state to unity at time zero, and as time elapses, the excited state decays with a single exponential rate. At equilibrium, the excited state forms and is sustained by repetitive rounds of collision complex formation and decay. A second-chance pathway for collision complex formation occurs when a steady-state process delivers additional switches to the site of the reader before the excited state has decayed. The steady-state process is mechanical in the flagellar system modeled here, but the model is not specific for the form of work that sustains the steady-state.

In the comparable skeletal muscle model, the second-chance pathway is not driven by mechanical energy. The ATP hydrolysis cycle of myosin provides the steady-state required for the second-chance pathway. In the absence of ATP, the equilibrium pathway of muscle produces M by rigor binding of myosin. Although stable, the rigor bond cycles too slowly to allow movement. In a second-chance pathway, the breaking of the rigor bond by ATP releases one myosin from tropomyosin, only to be replaced by a second myosin that has hydrolyzed ATP before M has decayed [25]. Hence, chemical energy drives the second-chance mechanism of skeletal muscle.

The model described here requires two states for the rotor subunits and two states for the motor. The simplest flagella model would comprise only the CW and CCW states [3], which is consistent with cooperative activation by CheYP, if a concerted allosteric model applies [18]. The conformational spread version of allosteric theory includes substates of a 34-member ring undergoing randomly ordered conformational changes, but the resultant intermediates of the stator during a switching event could be considered a transition rather than a collection of distinct states [23], [24]. Hence, an allosteric model has inherently fewer states than a second-chance model. Furthermore, the allosteric model, but not the second-chance model, is consistent with unidirectional motor units. However, for motor units that are bidirectional, second-chance signal transduction provides a mechanism.

Cooperative flagellar switching without ligand binding has been observed by over-expressing flagellar subunits locked in either the ligand-bound or ligand-free functional state [27]. In our model, the ligand simply flips the switch complex between two conformations, and the conformational change could occur independently of ligand, e.g., by lowering the temperature [26]. In a previous experiment [27], if “always-on” and “always off” switch complexes were to insert randomly in a given rotor, the dependence of the output on the composition of the rotor (fraction of on versus off switch complexes) would be as cooperative as the ligand-dependent output because our model predicts that stochastic ligand binding determines the fractions of native on and off switch complexes (Fig. 1). By an allosteric mechanism, ligand-induced conformational changes are transmitted between adjacent subunits, and a rotor composed of randomly spaced switch complexes locked in alternative conformations disrupts the spread of conformational changes required for a cooperative output.

Although at least 10 motor units form a stator complex, we chose to fit cooperative CW bias data assuming an ensemble of *n* = 5 motor units for two reasons. First, CW bias data have been fit with a Hill coefficient of ∼10 [20], which is consistent with our model given *α* = 1. Second, to explore α>1, it is necessary to reduce the value of *n*. However, the possibility of a variable number of motor units acting at a particular moment on a rotor cannot be excluded, given the rapid turnover of motor units in a stator [14].

Based on *α* alone, one would expect a direct relationship between rotor speed and measures of CW bias; however, the relationship is nearly constant over a broad range [28]. This draws attention to the maximum rate by which a collision complex can form while the rotor is in motion (*k*_r+_; Fig. 1). If the rate of collision complex formation were to be limited by a chemical step, *k*_r+_ could be less than the rotor rate, *i.e*., *k*_r+_≤*k*_r_.. In contrast, a collision complex must decay back to ground state by the rate of the rotor, *i.e*., *k*_r−_ = *k*_r_. Under circumstances, where the ratio *k*_r+_/*k*_r−_ is decreasing owing to constant *k*_r+_ and increasing *k*_r_, CW bias should asymptotically approach a minimum, which is what was observed [28].

Although the proposed model has two explicit pathways, we constrained the simulation to a single combination pathway for simplicity. The observed fit for *α* = 1 can be explained, in part, by the mathematical simplification of the analytical expression; for all other values of *α*, the analytical expression has a second-order dependence on ligand. When *α* = 1, the two pathways contribute equally (Eq. 2.8). For all other values of *α*, the simulation does not take into consideration the unequal contributions of the two pathways. We observe a discrepancy between simulated and predicted results for *α* = 2, which should become increasingly severe as *α* is increased. The simulation presented here must be refined to account for two pathways and to fully test the proposed molecular mechanism for all values of *α* as well as for additional characteristics of the flagellar motor.

Although it is artificial, constraining the simulation to prevent a ground state event between pulses may be justified by the characteristics of the rotor and stator. A high duty ratio by a motor unit [17] could sustain the excited state for the duration of a pulse. In addition, the stoichiometry of a rotor is 34 FliM subunits coupled with 26 FliG subunits [10], [11], from which a ligand-induced structural change in one FliM subunit could span two adjacent FliG subunits. In this way, the same ligand binding event could be “carried-over” during the transition between switch complexes arriving at a motor unit. Alternatively, a motor unit that spans multiple switch complexes could reduce *k*_−1_ (Fig. 1) during the transition period by inducing the same conformation in the approaching switch complex.

How does cooperativity improve performance? Considering only the average output, the damped output of ensemble motor units relative to the input of a single component unit (Fig. 3) is a clear benefit for achieving a smooth swimming motion of the flagella. In a larger context, cooperativity may be essential when the dynamic range of intracellular ligand concentration is constrained by adaptation [29]. Indeed, the flagellar switching mechanism may operate within a narrow range of CheYP concentration owing to a highly regulated signal transduction pathway, cf. [24]. An ultrasensitive response can be achieved with the concerted action of 10 motor units, given the Hill equation [19]. For *α*>1 (Eq. 2.14), similar sensitivity to ligand can be achieved with fewer motor units in an ensemble (Fig. 2B). Our simulation suggests that an orchestrating event such as torque could synchronize the switching of an ensemble from moment to moment. Finally, a theoretical physical limit to ligand sensitivity has been established for non-interacting sites of arbitrary linear dimension [30], [31]. This limit was estimated to be within a factor of three of the actual switching sensitivity of a flagellar motor based on a calculation using the Hill equation [19]. It would be of interest to see whether the flagellar motor could operate even closer to the physical limit if *α* were greater than one.

## Supporting Information

Methods S1 **Look here for a detailed description of the simulation program, standards used in the simulation, and preliminary results.**
(PDF)

Figure S1 **Components of the simulation of a single motor.** Three subroutines are connected in sequence, namely, Switch (2), Reader (3), and Output (4), corresponding to functional elements of the model we propose. The pulse generator (1) was set to 1 for the pulse width (dwell time), 95% for the pulse period (dwell time interval), and arbitrary amplitude between 0 and 1. The outputs of each of the components are connected to a scope (5), which displays the results in program time.
(TIF)

Figure S2 **Diagram showing components of the Switch subroutine.** This subroutine receives a value between 0 and 1 from the pulse generator (Prob 1). A pseudo-random variable between 0 and 1 is generated with a built-in function ({S2}). If the value of the random number is less than or equal to the probability a ligand is bound (Prob 1), the output is 1; however, if the value is greater than Prob 1, the output is 0. The ground ({S1}) caps an unused port of ({S2}). Data type conversion between Boolean and double precision is required by the program to maintain data storage compatibility with the next subroutine ({S4}).
(TIF)

Figure S3 **Diagram of the Reader subroutine.** The circuit composed of the integrator ({R1}) and a constant ({R2}) generates an exponential decay from an initial value of 1. A built-in solver uses {R2} and the output of the previous time step to compute the integral for output from {R2} at the current time step. The initial state of {R1} is set to 1; the initial state is restored if the input (Reset) rises from bits 0 to 1 at the beginning of a new pulse. {R2} has the value of the inverse time constant (tau). The value of {R1} at the onset of a pulse is held constant for the duration of the dwell time ({R3}) while the integrator continues. Zero Order Hold block, {R3}, outputs a discrete value between 0 and 1 to a port for the next subroutine (Prob 1).
(TIF)

Figure S4 **Diagram showing components of the Output subroutine.** Data type conversion between Boolean and double precision is required by the program to maintain data storage compatibility with the previous subroutine ({O1}). Given input of 1, Switch Block ({O2}) passes 1 to output (Event 1). The value of the previous Event 1 is stored in Hold Block ({O3}). Regardless of the value of Prob 1, if {O3} has a value of bit 0, Switch Block ({O4}) outputs 0, which then passes to Event 1. For Prob 1<1 and {O3} equal bit 1, the value of Prob 1 passes from Switch Block ({O4}) to be evaluated at logic block {O5}, If Prob 1 is greater than or equal to a pseudo-random number generated by Function Block ({O6}), Event 1 receives bit 1. Otherwise, Event 1 receives bit 0.
(TIF)

Figure S5 **Output of uncorrected simulation.** The four records are simultaneous outputs of the components shown in diagrammatic form (Fig. 1S), namely, Pulse Generator and Switch, Reader, and Output subroutines. The probability of the excited state of the Reader subroutine rises to 1 when a value of 1 is received from the Switch subroutine. Although declining exponentially, discrete values of the excited state probability are seen as greater than zero (*, Motor) for dwell times after the stimulation (*, Switch). The lifetime of the excited state probability gives rise to bit 1 events from the Output routine during intervals with no stimulation from the Switch routine (record between dotted lines). Resurrection of an excited state event after a ground state event without stimulation (arrows, Output) contradicts a premise of our model, namely, an excited state requires coupling by a ligand bound switch complex. The program is shown in Fig. 4S corrects for this error.
(TIF)

Figure S6 **Output of simulation using a circuit that corrects for spurious output.** With additional logic code, the Output subroutine (Fig. 4S) filters out spurious resurrections (Fig. 5S), but does not terminate the simulated lifetime of the associated excited state probability. Although effective and expedient, this filtering solution does not fully conform to the workings of the model as described in the supplementary text.
(TIF)

Figure S7 **Diagram and sample output of the simulation program.** A. The Simulink program with five motor units (n = 5) shows the logic circuit that reverses the binary output of the previous sample time only when the vector of the motor routine outputs is exclusively 0 or 1. B. A sample record of dwell time pulses was collected from one motor unit and the ensemble of five motor units.
(TIF)

Figure S8 **Comparison of predicted and simulated CW bias in response to arbitrary decay rate.** The purpose is to identify a minimum simulated CW bias of a single motor unit given constant dwell time interval and ligand binding probability. Increasing the decay rate (τ^−1^) reduces the opportunity for a ligand binding event to stimulate the motor to the excited state, which is required for CW output. The CW bias, calculated for one motor (n = 1) using the M function (see below), is shown for three values of α. Conditions: The dwell time interval and ligand binding probability are set in the simulation to unity and 0.5 respectively. Each point represents the average output of 10,000 pulses (Fig. 1S). For simplicity, the coupling and ligand binding constants, *K*_0_ and *K*_L_, are set to unity. Given these conditions the M function for one motor unit simplifies to M = (1−M)(1+(α−1)M).
(TIF)
