# Supplementary material for: Second-Chance Signal Transduction Explains Cooperative Flagellar Switching
Source: PLoS One. 2012 Jul 23;7(7):e41098. doi: 10.1371/journal.pone.0041098 (PMC3402542; doi:10.1371/journal.pone.0041098)
Supplement: Methods S1 — Look here for a detailed description of the simulation program, standards used in the simulation, and preliminary results. (PDF) [file pone.0041098.s001.pdf]

Supplementary Methods

Simulation Program

We assembled a program using the visual software package Simulink (Mathworks, Natick, MA USA). Aside from offering a library of useful subroutines and functions, Simulink simplifies the task of programming and coordinating multiple processes operating at independent rates.

The program is based on a constant rate of interactions between switch complexes that bind ligand and a single motor unit (Fig. 1S). The constant rate of signals received by a motor is simulated by a pulse generator. The amplitude of a pulse is a variable that corresponds to the probability a ligand is bound (θ). The width of the pulse determines the time a switch complex dwells at the dwell site of a motor. The period of the pulse is the fraction of the width in which the amplitude is not zero, but the output is held constant. A single value between 0 and 1 from the pulse generator is processed, by a sequence of subroutines (Fig. 1S), to a bit that is recorded for visual display and stored in a vector array for further analysis. As a conceptual aid, we divided one sequential program into three subroutines with logical relationships to our model.

The Switch subroutine simulates stochastic transitions between two conformations of a single switch complex. Based on a probability received from the pulse generator, the Switch subroutine simulates a stochastic binding event and outputs the result as bit 0 for conformation u or bit 1 for conformation U (Fig. 2S). The bit is held for the duration of the pulse.

The Reader subroutine simulates the decay of the excited state of a motor unit (Fig. 3S). The time constant (τ) is an experimental variable. Initially the integrator of the subroutine is set to 1, representing the probability that a motor unit is excited. The probability declines with a single exponential rate, τ^{-1}. If the value 1 is received from the Switch subroutine, the integrator is reset to probability 1. The output is held at a discrete value for the duration of the pulse.

The main function of the Output subroutine is to simulate a stochastic event based on the probability of the excited state (Fig. 4S). It is helpful to note that the input probability can be 1 only if ligand binding simulated by the Signal subroutine is bit 1 (see above). If probability 1 is received, the output of the Output subroutine will always be bit 1, which conforms to the

constraint that coupling is 100% effective. Otherwise, the output bit is simulated based on the input probability, except for the following prevailing condition.

If during the previous dwell time the output was bit 0 (ground state), the present output cannot be bit 1. According to the model, formation of the excited state of a motor unit requires energy coupling with a ligand-bound switch complex, and the lifetime of the excited state probability is intrinsic to the motor unit until the moment a ground state event occurs. Thereafter, the probability of an excited state event should be zero until a fresh excitation event takes place. However, if uncorrected, the simulation program allows an excited state event to take place without being stimulated (Fig. 5S). We addressed this problem by including a block that stores the previous bit (Fig. 4S); if bit 0 is the previous state of the motor unit and input probability is less than 1, then the current output is set to bit 0. Functional values of the Output subroutine are summarized in Table 1S.

Our solution to correct the output record is effective (Fig. 6S), but with a cautionary note. Rather than terminating the excited state, the program allows the excited state to decay after a ground state event. Spurious excited state events are filtered before output. Hence, the output of the simulation is faithful to the model, but not the internal state of the program.

To achieve an all-or-none output, the individual output bits of all ensemble motors are compared in a logic circuit to the bit value of the most recent ensemble output (Panel A; Fig 7S). The motor subroutines are as described above. All receive a simultaneous pulse, which carries the a given amplitude (probability of ligand binding). The most recent output is stored as bit 1 (CW) or bit 0 (CCW). For an arbitrary pulse, the program first compares the bits from the individual motor units using the logic AND function for identity. If the comparison meets the AND criteria, the program assigns a bit corresponding to the bit of the motor units and compares the assigned bit to the bit stored from the most recent output. If the second comparison does not meet the AND criteria, the output is switched from the stored bit value to the opposite bit value. A record from any one of the ensemble motor units shows many more reversals than the output of the ensemble (Panel B; Figure 7S).

Simulation Standards

The θ was determined for arbitrary [L] from the standard hyperbola with K_L equal to 3.7 μM. In a Simulink program, the dwell time for a switch complex was held constant (1 unit/event), and θ was the amplitude of 10,000 pulses, where each pulse simulated a switch complex-motor interaction. CW Bias was the fraction of bit 1 events out of the total.

Preliminary Results

We determined conditions in which the output of the simulation approaches the CW bias predicted by α = 1 and α = 2. Given pulses of constant dwell time (1 unit/pulse), the simulation was conducted for variable τ^{-1} in a range 0.8-10 $units^{-1}$. For $\tau^{-1} > 4$ the simulated CW bias converged on the value predicted by the M function for α = 1 (Fig. 7S). Smaller decay rates were seen to increase CW bias (Fig. 7S), which is consistent with our model. We estimated that the CW bias simulated with $\tau^{-1} = 0.8$ was sufficiently close to the CW bias predicted by the M function for α = 2 (Fig. 7S).

Supplement Table

Table 1S. Truth table for the Output subroutine

Input Probability	Previous Output	Present Output
1	0 or 1	1
<1	1	0 or 1
<1	0	0

Supplement Figure Legends

Figure 1S. Components of the simulation of a single motor. Three subroutines are connected in sequence, namely, Switch (2), Reader (3), and Output (4), corresponding to functional elements of the model we propose. The pulse generator (1) was set to 1 for the pulse width (dwell time), 95% for the pulse period (dwell time interval), and arbitrary amplitude between 0 and 1. The outputs of each of the components are connected to a scope (5), which displays the results in program time.

Figure 2S. Diagram showing components of the Switch subroutine. This subroutine receives a value between 0 and 1 from the pulse generator (Prob 1). A pseudo-random variable between 0 and 1 is generated with a built-in function ({S2}). If the value of the random number is less than or equal to the probability a ligand is bound (Prob 1), the output is 1; however, if the value is greater than Prob 1, the output is 0. The ground ({S1}) caps an unused port of ({S2}). Data type conversion between Boolean and double precision is required by the program to maintain data storage compatibility with the next subroutine ({S4}).

Figure 3S. Diagram of the Reader subroutine. The circuit composed of the integrator ({R1}) and a constant ({R2}) generates an exponential decay from an initial value of 1. A built-in solver uses {R2} and the output of the previous time step to compute the integral for output from {R2} at the current time step. The initial state of {R1} is set to 1; the initial state is restored if the input (Reset) rises from bits 0 to 1 at the beginning of a new pulse. {R2} has the value of the inverse time constant (tau). The value of {R1} at the onset of a pulse is held constant for the duration of the dwell time ({R3}) while the integrator continues. Zero Order Hold block, {R3}, outputs a discrete value between 0 and 1 to a port for the next subroutine (Prob 1).

Figure 4S. Diagram showing components of the Output subroutine. Data type conversion between Boolean and double precision is required by the program to maintain data storage compatibility with the previous subroutine ({O1}). Given input of 1, Switch Block ({O2}) passes 1 to output (Event 1). The value of the previous Event 1 is stored in Hold Block ({O3}). Regardless of the value of Prob 1, if {O3} has a value of bit 0, Switch Block ({O4}) outputs 0, which then passes to Event 1. For Prob 1 < 1 and {O3} equal bit 1, the value of Prob 1 passes from Switch Block ({O4}) to be evaluated at logic block {O5}, If Prob 1 is greater than or equal to a pseudo-random number generated by Function Block ({O6}), Event 1 receives bit 1. Otherwise, Event 1 receives bit 0.

Figure 5S. Output of uncorrected simulation. The four records are simultaneous outputs of the components shown in diagrammatic form (Fig. 1S), namely, Pulse Generator and Switch, Reader, and Output subroutines. The probability of the excited state of the Reader subroutine rises to 1 when a value of 1 is received from the Switch subroutine. Although declining exponentially, discrete values of the excited state probability are seen as greater than zero (*, Motor) for dwell times after the stimulation (*, Switch). The lifetime of the excited state probability gives rise to bit 1 events from the Output routine during intervals with no stimulation from the Switch routine (record between dotted lines). Resurrection of an excited state event after a ground state event without stimulation (arrows, Output) contradicts a premise of our model, namely, an excited state requires coupling by a ligand bound switch complex. The program is shown in Fig. 4S corrects for this error.

Figure 6S. Output of simulation using a circuit that corrects for spurious output. With additional logic code, the Output subroutine (Fig. 4S) filters out spurious resurrections (Fig. 5S), but does not terminate the simulated lifetime of the associated excited state probability. Although effective and expedient, this filtering solution does not fully conform to the workings of the model as described in the supplementary text.

Figure 7S. Diagram and sample output of the simulation program. A. The Simulink program with five motor units (n = 5) shows the logic circuit that reverses the binary output of the previous sample time only when the vector of the motor routine outputs is exclusively 0 or 1. B. A sample record of dwell time pulses was collected from one motor unit and the ensemble of five motor units.

Figure 8S. Comparison of predicted and simulated CW bias in response to arbitrary decay rate. The purpose is to identify a minimum simulated CW bias of a single motor unit given constant dwell time interval and ligand binding probability. Increasing the decay rate (τ^{-1}) reduces the opportunity for a ligand binding event to stimulate the motor to the excited state, which is required for CW output. The CW bias, calculated for one motor (n = 1) using the M function (see below), is shown for three values of α. Conditions: The dwell time interval and ligand binding probability are set in the simulation to unity and 0.5 respectively. Each point represents the average output of 10,000 pulses (Fig. 1S). For simplicity, the coupling and ligand binding constants, K_0 and K_L, are set to unity. Given these conditions the M function for one motor unit simplifies to $M = (1 - M)(1 + (\alpha - 1)M)$.
