# Supplementary figures and images for: Second-Chance Signal Transduction Explains Cooperative Flagellar Switching
Source: PLoS One. 2012 Jul 23;7(7):e41098. doi: 10.1371/journal.pone.0041098 (PMC3402542; doi:10.1371/journal.pone.0041098)

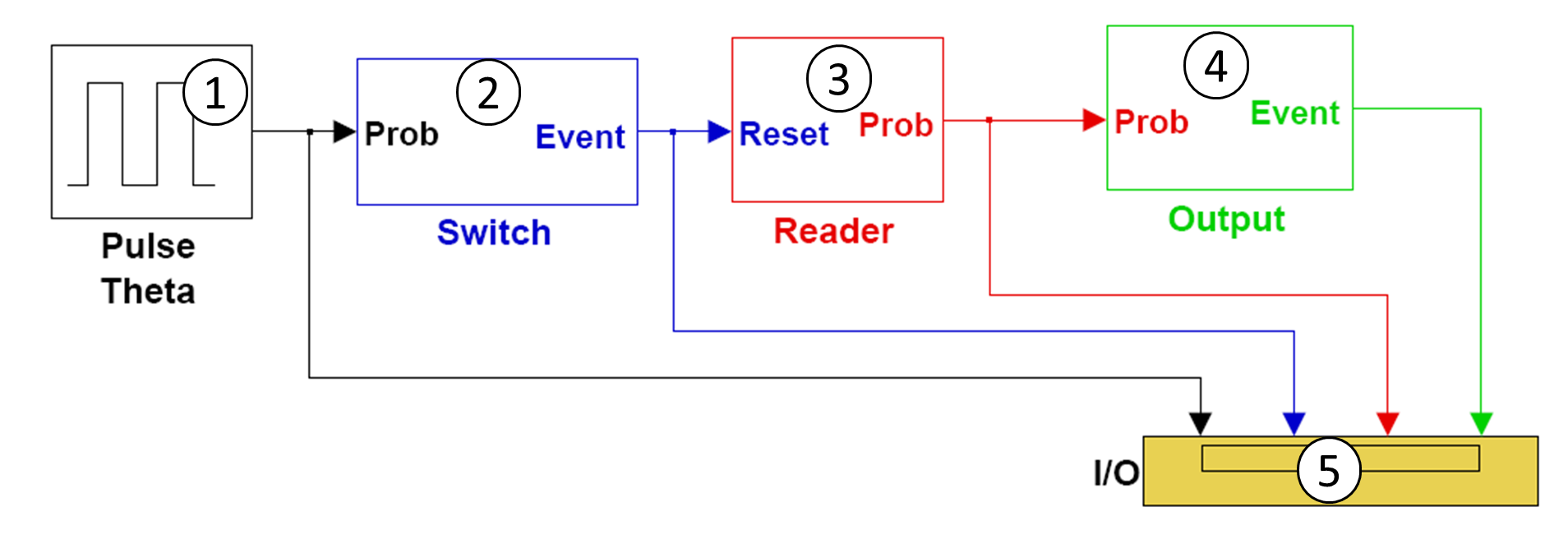

Supplement: Figure S1 — Components of the simulation of a single motor. Three subroutines are connected in sequence, namely, Switch (2), Reader (3), and Output (4), corresponding to functional elements of the model we propose. The pulse generator (1) was set to 1 for the pulse width (dwell time), 95% for the pulse period (dwell time interval), and arbitrary amplitude between 0 and 1. The outputs of each of the components are connected to a scope (5), which displays the results in program time. (TIF) [file pone.0041098.s002.tif]

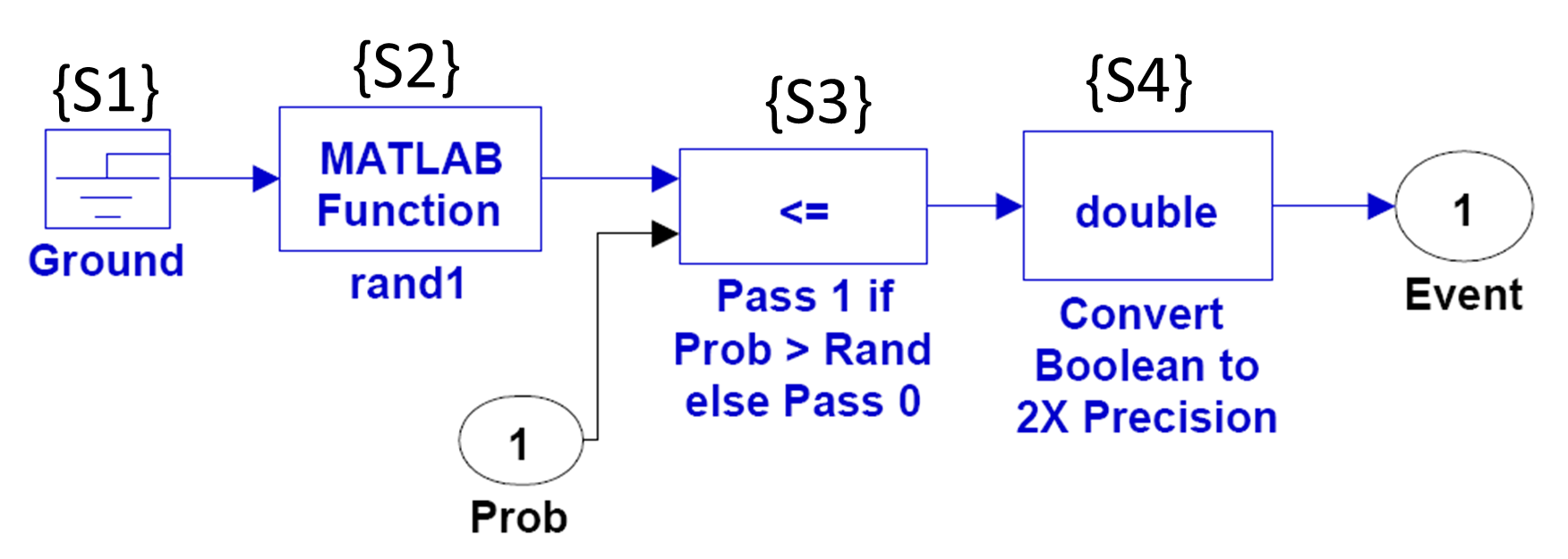

Supplement: Figure S2 — Diagram showing components of the Switch subroutine. This subroutine receives a value between 0 and 1 from the pulse generator (Prob 1). A pseudo-random variable between 0 and 1 is generated with a built-in function ({S2}). If the value of the random number is less than or equal to the probability a ligand is bound (Prob 1), the output is 1; however, if the value is greater than Prob 1, the output is 0. The ground ({S1}) caps an unused port of ({S2}). Data type conversion between Boolean and double precision is required by the program to maintain data storage compatibility with the next subroutine ({S4}). (TIF) [file pone.0041098.s003.tif]

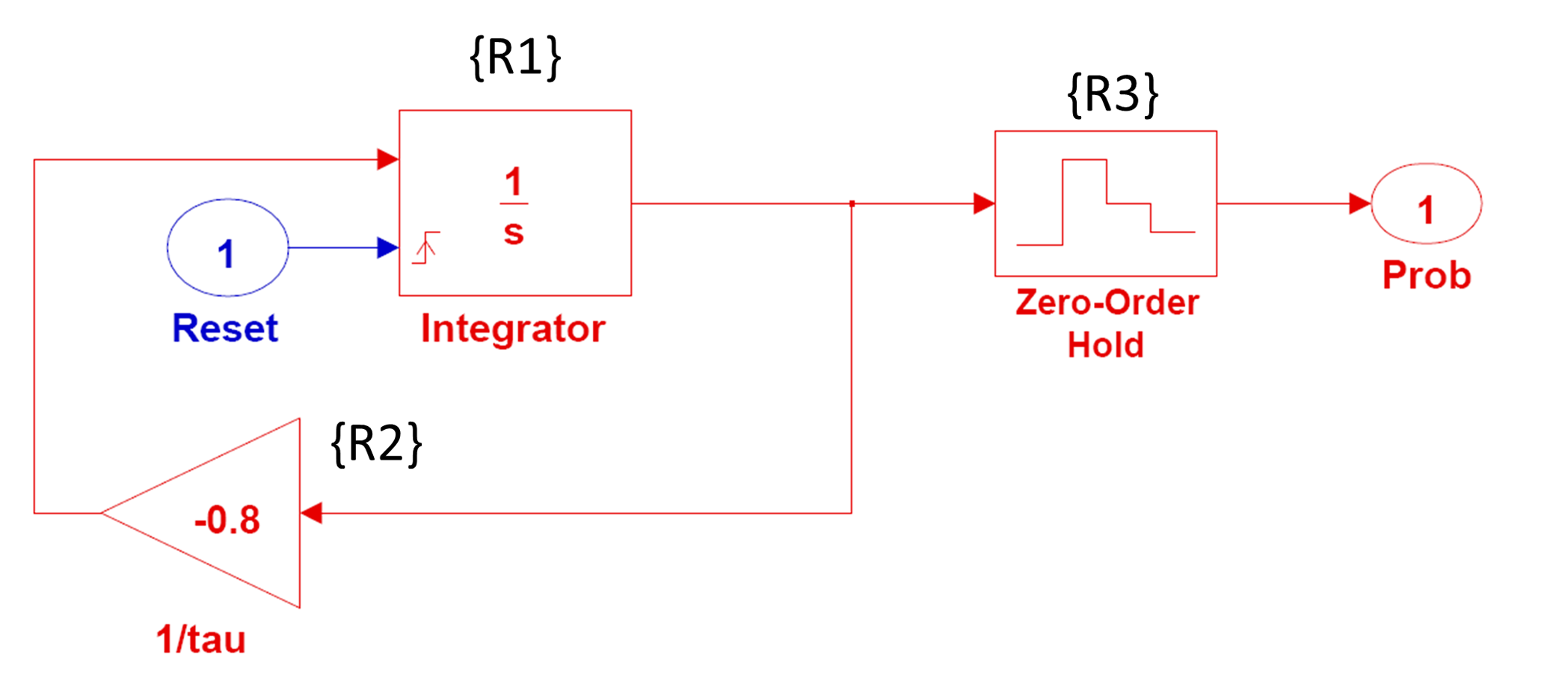

Supplement: Figure S3 — Diagram of the Reader subroutine. The circuit composed of the integrator ({R1}) and a constant ({R2}) generates an exponential decay from an initial value of 1. A built-in solver uses {R2} and the output of the previous time step to compute the integral for output from {R2} at the current time step. The initial state of {R1} is set to 1; the initial state is restored if the input (Reset) rises from bits 0 to 1 at the beginning of a new pulse. {R2} has the value of the inverse time constant (tau). The value of {R1} at the onset of a pulse is held constant for the duration of the dwell time ({R3}) while the integrator continues. Zero Order Hold block, {R3}, outputs a discrete value between 0 and 1 to a port for the next subroutine (Prob 1). (TIF) [file pone.0041098.s004.tif]

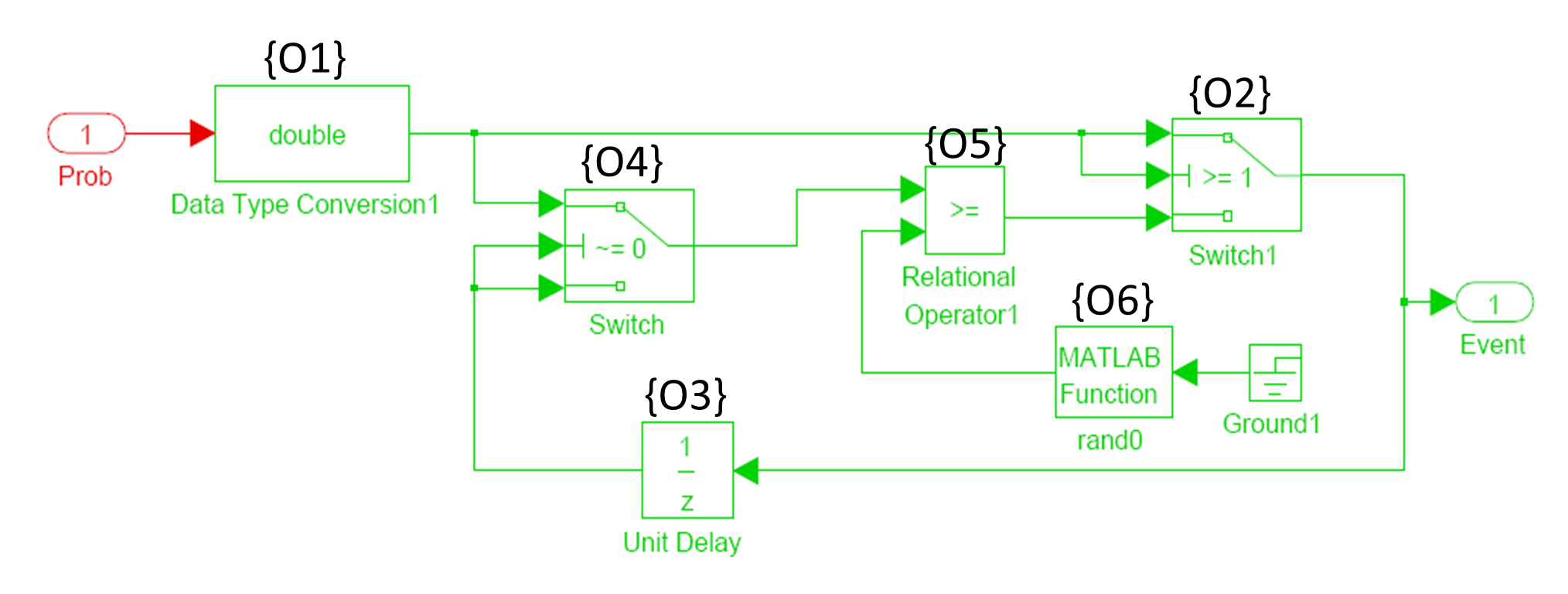

Supplement: Figure S4 — Diagram showing components of the Output subroutine. Data type conversion between Boolean and double precision is required by the program to maintain data storage compatibility with the previous subroutine ({O1}). Given input of 1, Switch Block ({O2}) passes 1 to output (Event 1). The value of the previous Event 1 is stored in Hold Block ({O3}). Regardless of the value of Prob 1, if {O3} has a value of bit 0, Switch Block ({O4}) outputs 0, which then passes to Event 1. For Prob 1<1 and {O3} equal bit 1, the value of Prob 1 passes from Switch Block ({O4}) to be evaluated at logic block {O5}, If Prob 1 is greater than or equal to a pseudo-random number generated by Function Block ({O6}), Event 1 receives bit 1. Otherwise, Event 1 receives bit 0. (TIF) [file pone.0041098.s005.tif]

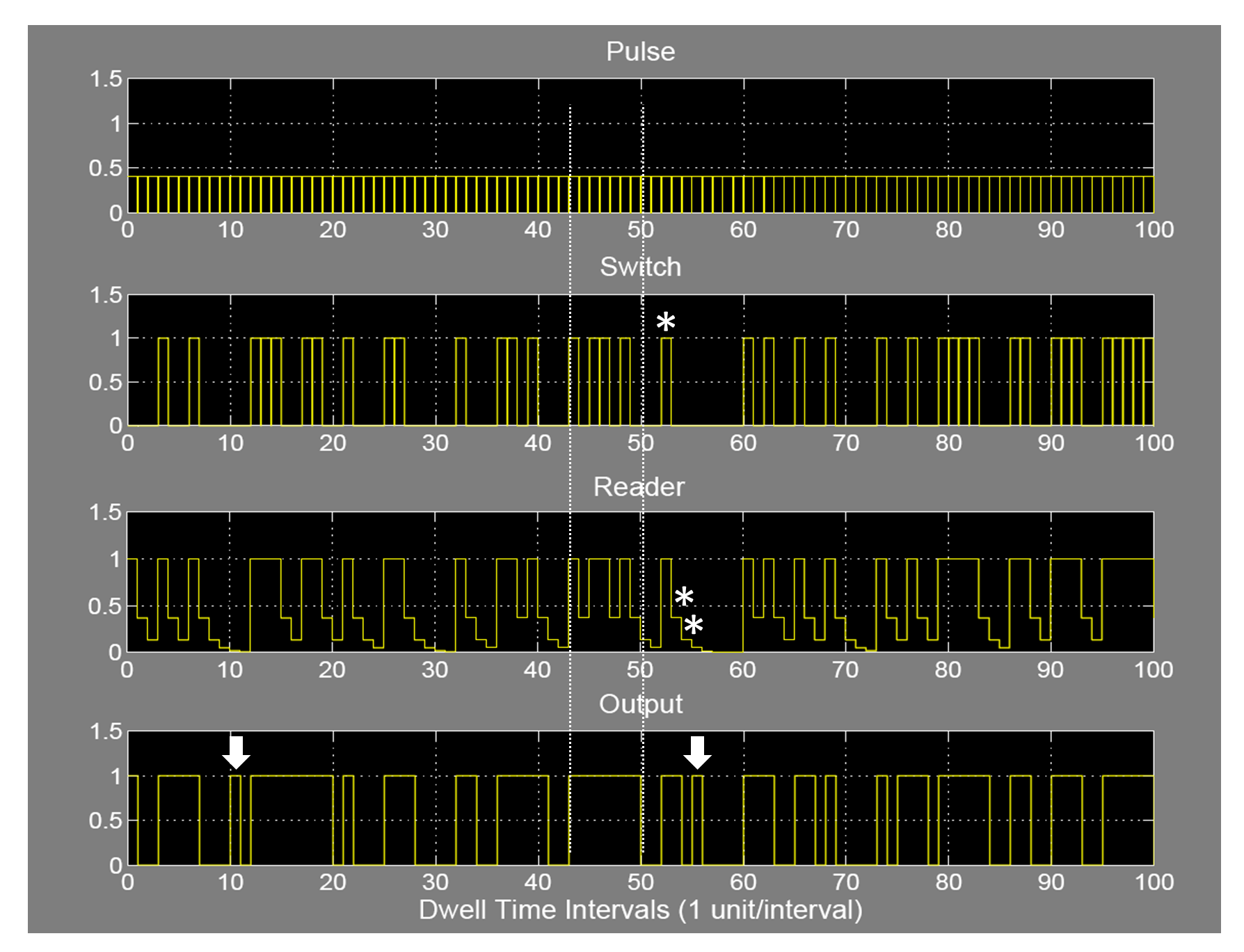

Supplement: Figure S5 — Output of uncorrected simulation. The four records are simultaneous outputs of the components shown in diagrammatic form (Fig. 1S), namely, Pulse Generator and Switch, Reader, and Output subroutines. The probability of the excited state of the Reader subroutine rises to 1 when a value of 1 is received from the Switch subroutine. Although declining exponentially, discrete values of the excited state probability are seen as greater than zero (*, Motor) for dwell times after the stimulation (*, Switch). The lifetime of the excited state probability gives rise to bit 1 events from the Output routine during intervals with no stimulation from the Switch routine (record between dotted lines). Resurrection of an excited state event after a ground state event without stimulation (arrows, Output) contradicts a premise of our model, namely, an excited state requires coupling by a ligand bound switch complex. The program is shown in Fig. 4S corrects for this error. (TIF) [file pone.0041098.s006.tif]

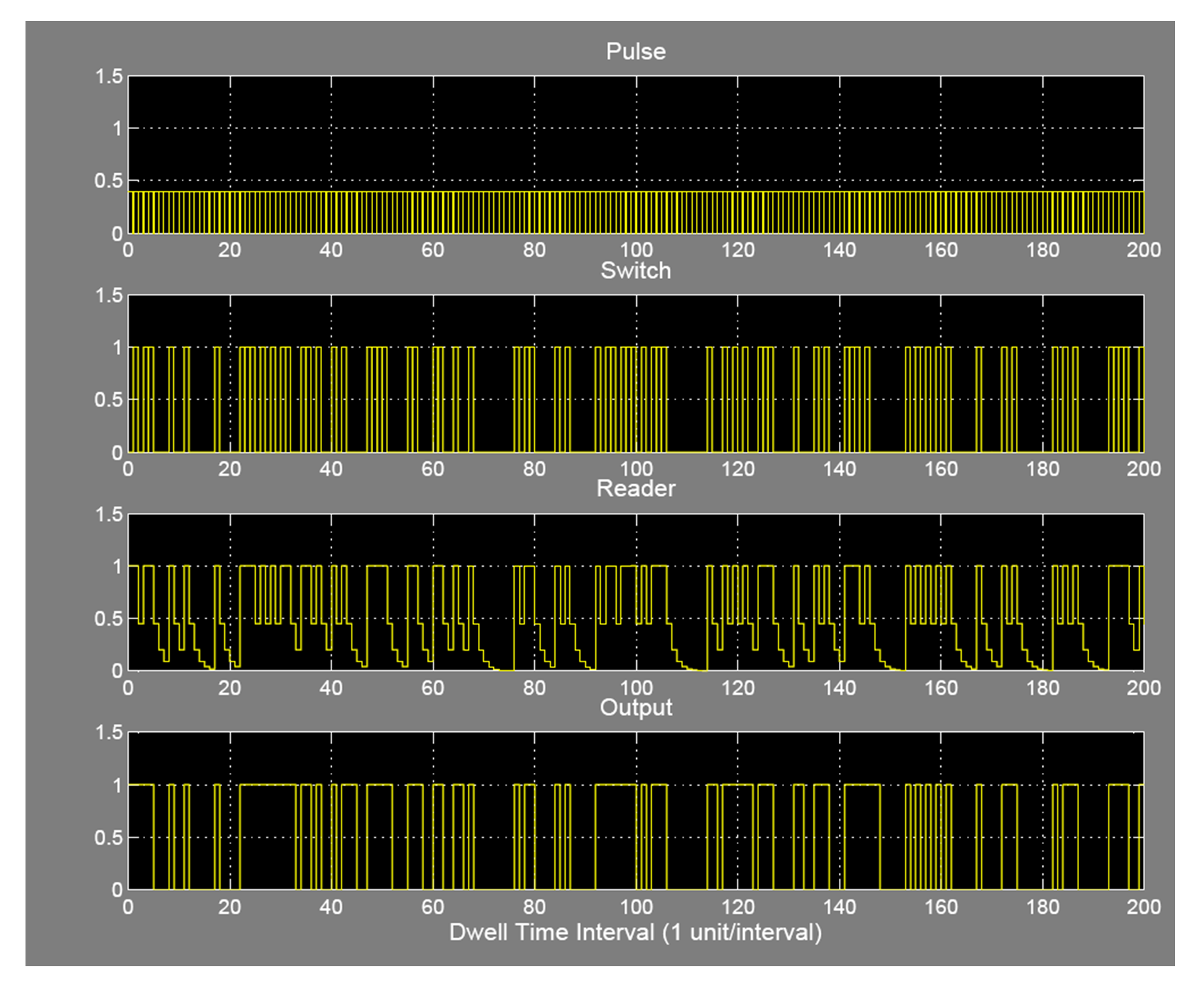

Supplement: Figure S6 — Output of simulation using a circuit that corrects for spurious output. With additional logic code, the Output subroutine (Fig. 4S) filters out spurious resurrections (Fig. 5S), but does not terminate the simulated lifetime of the associated excited state probability. Although effective and expedient, this filtering solution does not fully conform to the workings of the model as described in the supplementary text. (TIF) [file pone.0041098.s007.tif]

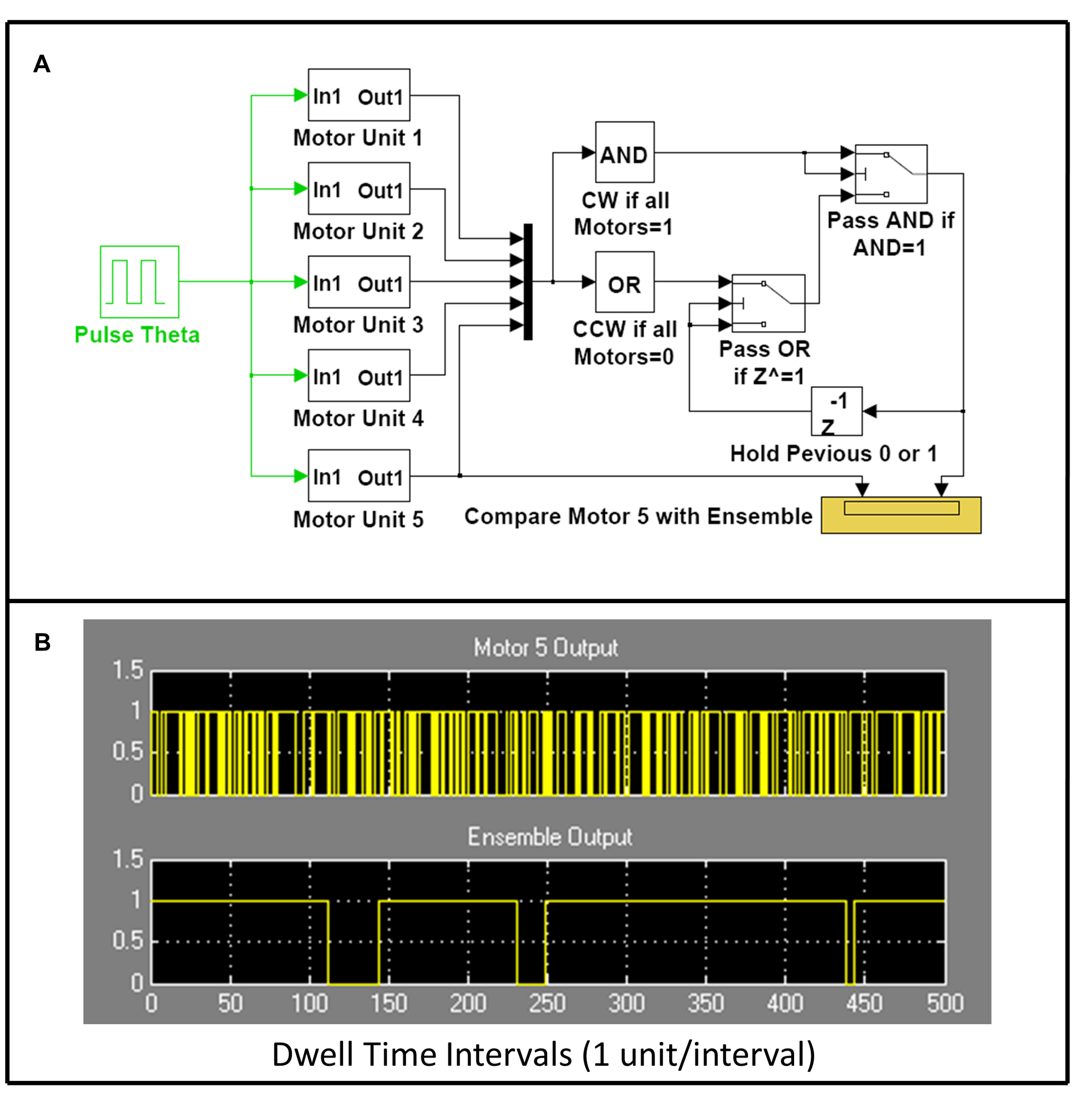

Supplement: Figure S7 — Diagram and sample output of the simulation program. A. The Simulink program with five motor units (n = 5) shows the logic circuit that reverses the binary output of the previous sample time only when the vector of the motor routine outputs is exclusively 0 or 1. B. A sample record of dwell time pulses was collected from one motor unit and the ensemble of five motor units. (TIF) [file pone.0041098.s008.tif]

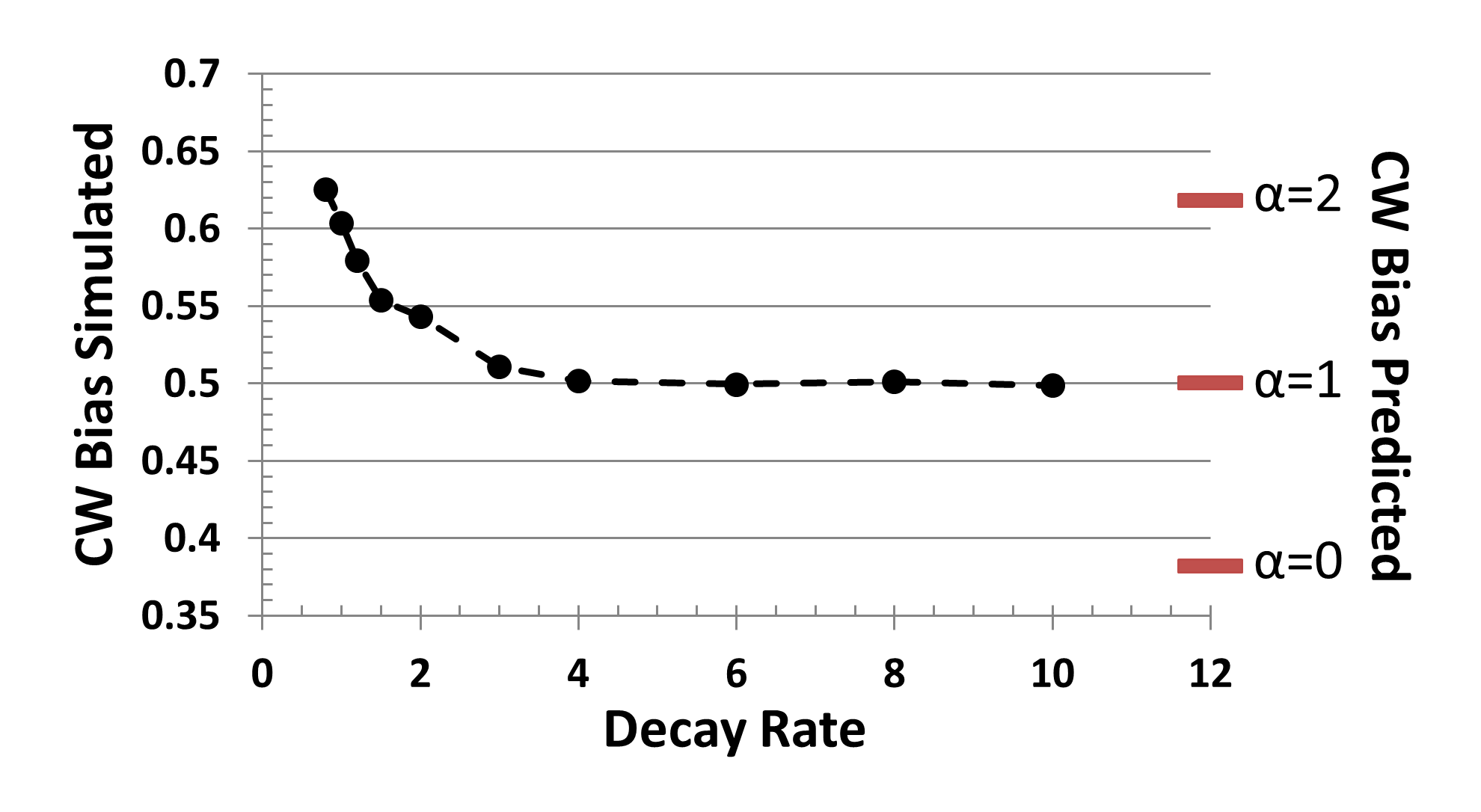

Supplement: Figure S8 — Comparison of predicted and simulated CW bias in response to arbitrary decay rate. The purpose is to identify a minimum simulated CW bias of a single motor unit given constant dwell time interval and ligand binding probability. Increasing the decay rate (τ−1) reduces the opportunity for a ligand binding event to stimulate the motor to the excited state, which is required for CW output. The CW bias, calculated for one motor (n = 1) using the M function (see below), is shown for three values of α. Conditions: The dwell time interval and ligand binding probability are set in the simulation to unity and 0.5 respectively. Each point represents the average output of 10,000 pulses (Fig. 1S). For simplicity, the coupling and ligand binding constants, K 0 and K L, are set to unity. Given these conditions the M function for one motor unit simplifies to M = (1−M)(1+(α−1)M). (TIF) [file pone.0041098.s009.tif]
